# Multilayer porous silicon diffraction gratings operating in the infrared

**DOI:** 10.1186/1556-276X-7-645

**Published:** 2012-11-24

**Authors:** Meifang Lai, Gayathri M Sridharan, Giacinta Parish, Shanti Bhattacharya, Adrian Keating

**Affiliations:** 1 School of Mechanical and Chemical Engineering, University of Western Australia, 35 Stirling Hwy, Crawley, Western Australia, 6009, Australia; 2 Department of Electrical Engineering, IIT Madras, Chennai, 600036, India; 3 School of Electrical, Electronic and Computer Engineering, University of Western Australia, 35 Stirling Hwy, Crawley, Western Australia, 6009, Australia

**Keywords:** Porous silicon, Diffraction grating, Diffraction efficiency, Photolithography, Etching, Infrared, Sensor

## Abstract

Transmission diffraction gratings operating at 1,565 nm based on multilayer porous silicon films are modeled, fabricated, and tested. Features down to 2 μm have been patterned into submicron-thick mesoporous films using standard photolithographic and dry etching techniques. After patterning of the top porous film, a second anodization can be performed, allowing an under-layer of highly uniform porosity and thickness to be achieved. High transmission greater than 40% is measured, and modeling results suggest that a change in diffraction efficiency of 1 dB for a 1% change in normalized refractive index can be achieved. Preliminary measurement of solvent vapor shows a large signal change from the grating sensor in agreement with models.

## Background

Diffraction gratings built from porous silicon (PS) have enormous potential to produce highly sensitive and rapid detection of analytes [1]. Planar gratings respond to changes in the near surface refractive index, requiring only shallow analyte infiltration compared with PS sensors made from microcavities and multilayer film stacks. Thin sensing layers have short analyte diffusion times enabling grating-based sensors to have fast response and reset times. PS-based grating sensors have previously been made using pre-etched silicon [2], direct laser writing [3,4], imprinting [5,6], and holography [7]. However, pre-etched silicon cannot achieve uniform layers, laser writing is slow, imprinting makes it difficult to control the optical properties of PS, and holographic methods have limited control of the patterns which can be produced. Further, the best results to date [5] have reported a measurable index change of only 3% which required measurement of the diffraction efficiency to around 10^−5^. In some cases, optical layers have been fabricated under the grating either as an uncontrolled result of the fabrication process [2,5] or by design [4,6]. Such layers could allow complex structures such as 2D photonic crystal structures to be created [6]; however, high-resolution feature definition of the patterned PS layers over well-defined multilayer PS optical films has not yet been achieved.

**Figure 1:**
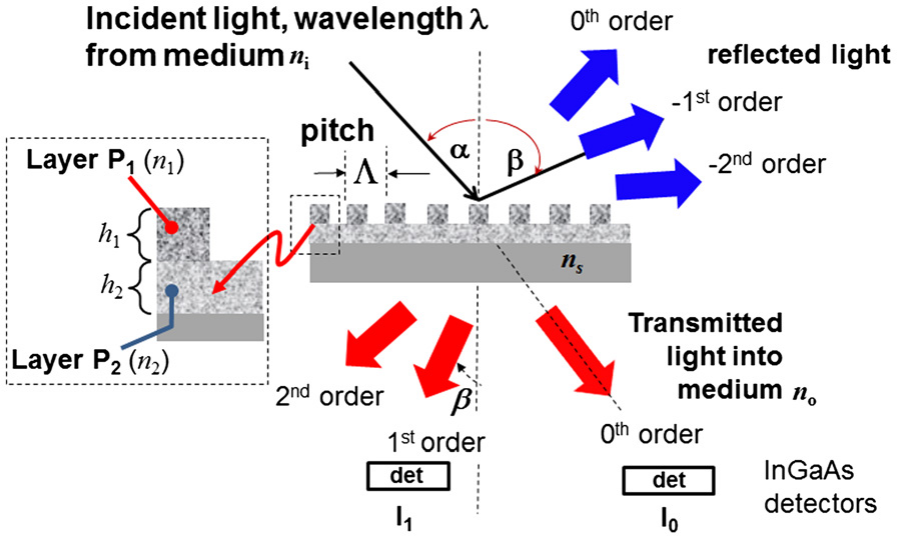
Diffraction grating showing the porous silicon multilayers and defined grating equation variables.

The method of sensing using a diffraction grating is based on changes to the diffraction orders caused by refractive index changes in the film. The grating equation defines the diffraction and is given by the equation below [8]:

(1) $$n_{i}\text{sin}\left(\alpha \right)+n_{0}\text{sin}\left(\beta \right)=m\frac{\lambda }{\Lambda }\text{,}$$

where *Λ* is the grating pitch, *m* the diffraction order (*m* = 0, ±1, ±2,.), *n*_i_ and *α* are the medium index and angle of the incident beam, respectively, and *n*_0_ and *β* are the medium index and angles of the *m*th order diffracted beam, respectively. These variables are shown in the grating illustrated in Figure 1. An important observation from the grating equation is that the angle of the diffracted orders is independent of the analyte (*n*_i_) refractive index when the incident angle *α* = 0. Under this condition, refractive index changes in the analyte surrounding the grating do not affect the position of the detectors measuring the power of the diffracted orders. Analyte index changes, however, do alter the power in each diffraction order, providing a simple means of signal detection compared with spectral measurements of film optical thickness or resonance dips in microcavity reflectance.

This work presents a pathway to create multilayered patterned features on porous films containing nano-sized pores, enabling high surface area, low loss infrared (IR) photonic crystal structures to be created. We present modeled and fabricated grating sensors based on porous silicon operating in transmission at *λ* = 1,565 μm. The gratings are fabricated on PS by combining our previously developed techniques which allowed standard photolithography be used [9] along with low roughness dry etching conditions [10]. This is the first time these microelectronic compatible techniques have been combined to achieve high-resolution features of uniform porosity and thickness, with all masking layers subsequently removed from the PS without film degradation. We also present a method for creating multilayer films with uniform porosity underneath the patterned porous silicon layer; the method is used to demonstrate improved grating performance. We fabricate a number of diffraction grating structures and compare the performance to models.

## Methods

A numerical model was developed to calculate the diffraction efficiencies using the rigorous coupled mode theory [11-13]. Diffraction efficiency is defined as the output power in each order normalized to the source power. The model included the grating shape, height, duty cycle, and refractive index of the diffraction grating. The refractive index (*n*_1_, *n*_2_) and heights (*h*_1_, *h*_2_) of the layers were designed to achieve operation in transmission at 1,565 nm. Height and index control of the gratings are important in obtaining high diffraction efficiencies [8]. Two structures were investigated (refer to Figure 1):

(1) Single-layer PS grating - a grating made from a single PS layer of porosity *P*_1_ (index *n*_1_ = 1.78, *h*_1_ = 512 nm, *h*_2_ = 0 nm), and

(2) Double-layer PS grating - a grating layer made from porosity *P*_1_ over a uniform layer of porosity *P*_2_ (index *n*_1_ = 1.78, *h*_1_ = 993 nm, *n*_2_ = 1.48, *h*_2_ = 262 nm).

All gratings were designed with a pitch of *Λ* = 4 μm and nominal ridge/groove ratio (duty cycle) of 50%.

Using our previously reported complementary metal-oxide semiconductor (CMOS) compatible photolithography [9] and reactive ion etching techniques [10], well-defined diffraction gratings were fabricated on highly uniform layers of porous silicon. Initially, a uniform layer of porous silicon was formed by means of electrochemical anodization on an area of 10 cm^2^ using 2-inch p-type double side-polished Si wafers with resistivity of 0.09 Ω·cm. A current of 1 mA/cm^2^ (*n*_1_ = 1.78) was used for the top porous layer. The anodization was performed using a conductive elastomer-backed contact in a single-tank anodization cell [14] with an electrolyte of 15% aqueous HF solution in 70% ethanol by volume. Once the desired layer thickness was achieved, the films were rinsed, dried using N_2_, and transferred to a rapid thermal annealer where a passivation of the layer was performed at 600°C for 6 min, based on our previous study [9]. The resulting films maintained their porosity (index) with less than 10% film thickness reduction, which was accounted for in the initial thickness estimation for films produced. The passivated films were subsequently suitable for standard photolithographic processing. A layer of diluted ProLIFT 100–16 (Brewer Science, Inc., MO, USA), a polymer based on *N*-methyl-2-pyrrolidone (NMP), was spun on the PS film at 6,000 rpm for 40 s and baked at 100°C for 2 min followed by 250°C for 1 min. The ProLIFT was applied prior to the photoresist layer and completely filled the pores, preventing photoresist seepage into the pores. The ProLIFT was not photodefinable and was easily removed from the pores using the same developer as the photoresist.

Subsequently, a layer of positive photoresist (AZ 6632, MicroChemicals GmbH, Ulm, Germany) was spun onto the ProLIFT-covered porous film at 6,000 rpm for 40 s, followed by soft baking at 110°C for 1 min. The photoresist was exposed with the grating mask using a standard UV mask aligner and developed using dilute AZ 400K (MicroChemicals) developer. The AZ 400K developer is a potassium-based buffered developer recommended for positive photoresists and is commonly used in the semiconductor fabrication industry. To obtain high contrast developing, the dilution ratio is one part of the AZ 400K mixed with four parts of deionised water. This results in an alkaline solution of approximate 1% KOH, at which concentration, as-fabricated porous silicon would normally dissolve in seconds. Our ProLIFT/AZ 6632-coated passivated films survived 80 s of development with less that 1% change in the optical thickness in the films. After development, the sample was hard-baked at 95°C for 5 min to prevent photoresist deformation during plasma etching.

After photolithography, the porous films were patterned using an inductively coupled plasma reactive ion etcher (ICP-RIE). The etch conditions producing a relatively vertical etching profile were determined to be as follows: CF_4_ flow rate of 31 sccm, CH_4_ flow rate of 3 sccm (percentage concentration of 9%), RF power of 200 W, ICP power of 400 W, chamber pressure of 80 mTorr, and substrate temperature of 20°C. After patterning of the top layer by RIE, the films were cleaned in acetone (for 5 min) and developer (for 10 s) to remove the positive photoresist and ProLIFT used to mask the PS film for the RIE. Complete removal of these polymers was confirmed by transmission Fourier transform infrared spectroscopy.

For the double-layer grating, after photoresist and ProLIFT striping, the films were re-immersed into the anodization cell. Upon immersion into the HF/ethanol solution, the oxide-rich passivation is removed, leaving a surface similar to as-fabricated PS which can undergo further anodization. A second low-index layer was formed at a current density of 10 mA/cm^2^. To achieve environmental and chemical stability for further processing, the samples could be repassivated by annealing in N_2_ at 600°C [9]; however, this step was not performed in these experiments.

**Figure 2:**
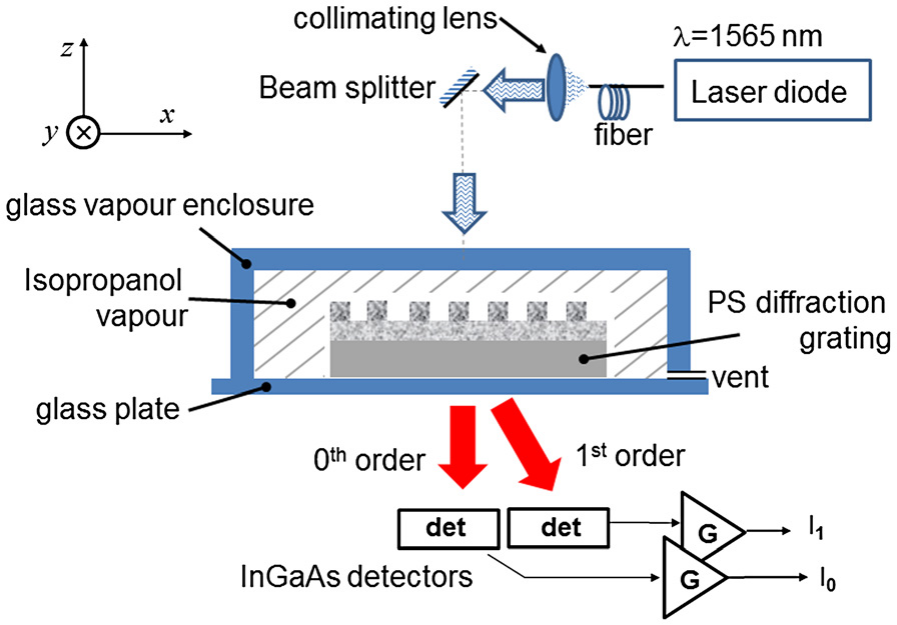
**Experimental setup used for measurement of the sensing performance of the PS grating.** The table is in the *xy*-plane indicated by the axes.

Diffraction grating characterization was performed using a detector whose position was controlled by an in-house built servo motor. Reflection measurements utilized a Si detector (10 × 0.5 mm^2^) and a 632-nm HeNe laser to illuminate the grating, while transmission measurements used a Ge detector (8 × 0.5 mm^2^) and a collimated 1,565-μm laser diode. Measurements of the sensing ability of the grating were performed using the setup illustrated in Figure 2. The sample was held on a glass plate in the *xy*-plane of the table, while the transmission through the grating was detected by two InGaAs detectors arranged to detect the 0th-order (*I*_0_) and 1st-order (*I*_1_) power. The solvent was introduced into the fixed volume glass enclosure which had a small opening to allow the vapor to slowly vent, while the powers (*I*_0_, *I*_1_) were recorded over time.

## Results and discussion

**Figure 3:**
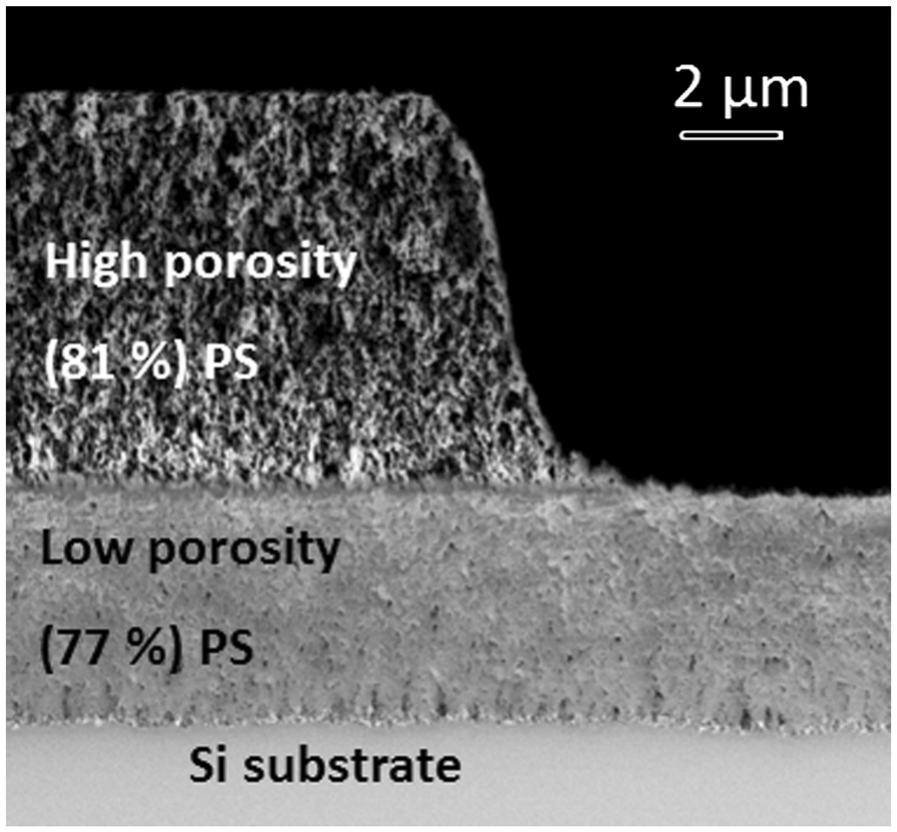
**A cross-sectional SEM image (beam voltage of 0.5 kV) of a double-layer PS film.** The first high-porosity layer was etched by ICP-RIE followed by low porosity film growth after the etch.

For large patterned features (>50 μm), the photolithographic process was based on ProLIFT 100–16 applied under standard spin conditions (6,000 rpm/40 s), resulting in a ProLIFT thickness of 350 nm [9]. Figure 3 shows an SEM micrograph of a sample fabricated by the multilayer patterning and re-anodization process. A layer of 5.65-μm thick high porosity (81%) porous silicon is initially formed and then patterned using CH_4_/CF_4_ in a RIE. The patterned PS layer shown here is slightly rounded due to minor deformation of the photoresist during RIE etching.

After the patterning of the first PS layer (*P*_1_), the sample was anodized again in the HF/ethanol solution with current density of 5 mA/cm^2^ for 15 min. The resultant second layer PS (*P*_2_) had a thickness of 3.6 μm and a porosity of 77%. The top interface of the *P*_2_ layer appears lower by around 150 nm (limited by the image resolution in Figure 3) in the region where no *P*_1_ layer is present, which was attributed to a slight over etch of the RIE into the silicon. A similar step of 150 nm is observed at the interface between the *P*_2_ layer and the silicon; however, the *P*_2_-layer thickness appears uniform throughout. This step in the *P*_2_-layer/Si interface was attributed to the transfer of the RIE surface profile into the underlying layer during anodization.

Previous observations have shown that the etch rate slows down by 2% to 3%/μm, and porosity gradients of around 5%/μm occur during anodization of lightly doped silicon [15]. These changes in etch rate and porosity occur at high current densities (typically >100 mA/cm^2^) [16] due to HF diffusion through the pores. At the low current densities and relatively thin layers used in this work, the change in etch rate and porosity due to HF diffusion is negligible. Another mechanism that could affect uniform porosity and etch rates is spatial current density variation in the *P*_2_ layer caused by the patterned *P*_1_ layer. Where the *P*_1_ layer porosity is low, or the layer very thick, spatial variation of the current density may become significant. However, since the conductivity of the HF is significantly greater than the carrier depleted, high porosity *P*_1_ layer shown in Figure 3, the potential at the Si-electrolyte surface is unaffected by the *P*_1_ layer. In the conditions used for anodization in this work, patterning of the *P*_1_ layer did not visibly affect the uniformity of the porosity or thickness in the P_2_ layer. Accurate control of the RIE process was the key in achieving the high level of interface flatness. With due consideration of the issues detailed above, multiple layers of porous films could be created underneath patterned structures with negligible loading effects from the pattern layers above them. Uniform porosity and interface layers are extremely important for high-quality photonic sensors and cannot be achieved by stamping [5], patterning of silicon followed by anodization [6], or masking of silicon followed by anodization [17].

Patterning of the 2-μm grating features demonstrated in this work initially failed because all the ProLIFT, including that under the photoresist, dissolved during developing due to the small dimensions of the mask. As feature sizes decrease, the allowable undercut must also be reduced to avoid delamination of the photoresist. To overcome this difficulty, ProLIFT 100–16 (16% solid) was diluted using NMP to ProLIFT 100–7.6 (7.6% solid) for the single layer PS grating and to ProLIFT 100–10.6 (10.6% solid) for the double layer PS grating, so as to be just sufficient to fill in all the pores. The significantly reduced thickness of the ProLIFT layer resulted in successful patterning of the *P*_1_ layer.

**Figure 4:**
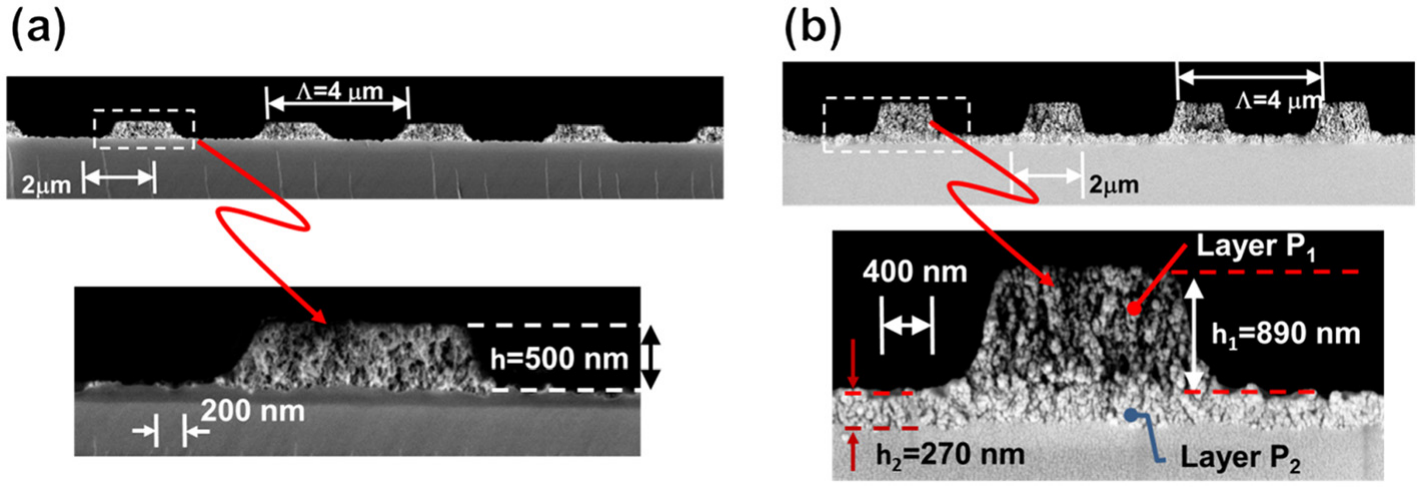
**A cross-sectional SEM image (beam voltage of 0.5 kV) of PS diffraction gratings.** Gratings were fabricated with a pitch of 4 μm; (**a**) single-layer grating of height *h*_1_ = 500 nm and (**b**) double-layer grating of heights *h*_1_ = 890 nm and *h*_2_ = 270 nm.

An SEM image of the fabricated PS diffraction gratings is shown in Figure 4. The single-layer grating is shown in Figure 4a, having a measured height of 500 nm, close to the design value of 512 nm. The patterned PS features have reproduced the grating mask extremely well. Feature definition for the double-layer grating shown in Figure 4b is slightly better than the single-layer grating; however, undercut during processing reduced the ridge/groove ratio of the grating from 50% (as designed) to 35%. Layer heights are very close to designed values. The grating optimized height (*h*_1_) is considerably larger than that reported using imprinting techniques [5]. These SEM images and corresponding height measurements validate the accuracy of the process to achieve the design specification, given that the samples have undergone thermal annealing, up to two anodizations, photolithography, and dry etching processes. All gratings show good quality diffraction of light under visual inspection.

**Figure 5:**
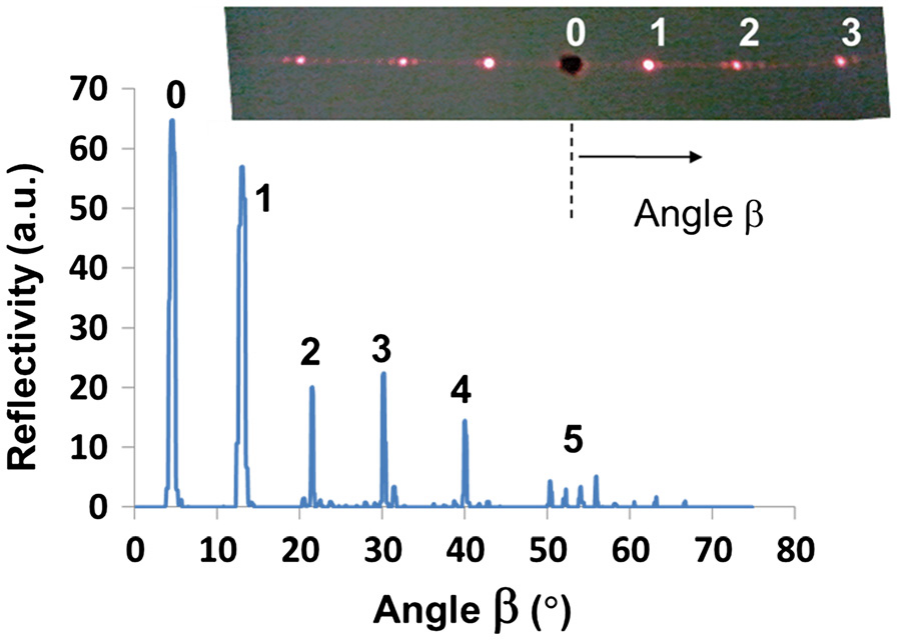
**Reflection measurement of the diffraction orders at *λ *= 632 nm from the single-layer grating.** Inset shows the image of the reflected orders on a screen with the incident light and 0th order passing through the same aperture.

Although designed for optical operation at 1,565 μm, initial diffraction measurements for the PS diffraction grating were obtained by reflecting a visible laser (HeNe laser at 632 nm) from the surface of the single-layer grating for comparison. The grating was angled at *α* = 4.5° relative to the incident laser beam to ensure that the 0th-order power could be measured. A large 0th-order power is expected at *λ* = 632 nm as the grating was not optimized at this wavelength. The measured reflected visible light diffraction spectrum is shown in Figure 5. The inset shows the light reflected off the sample and onto a screen (at *α* = 0°), where the central hole in the screen allowed the incident light to pass. The angular locations of the diffraction peaks obey the diffraction grating equation given in Equation 1. Both the inset and the measured data show considerable energy spread between each diffraction order which is not explained using the diffraction equation. These side lobes are largely attributed to the grating shape, non-optimum grating height (at *λ* = 632 nm), surface roughness, and interferometric effects from the Si interface. For sensing applications, scattering and optical interference lead to crosstalk between detectors which are designed to detect the power of specific orders as illustrated in Figure 1.

**Figure 6:**
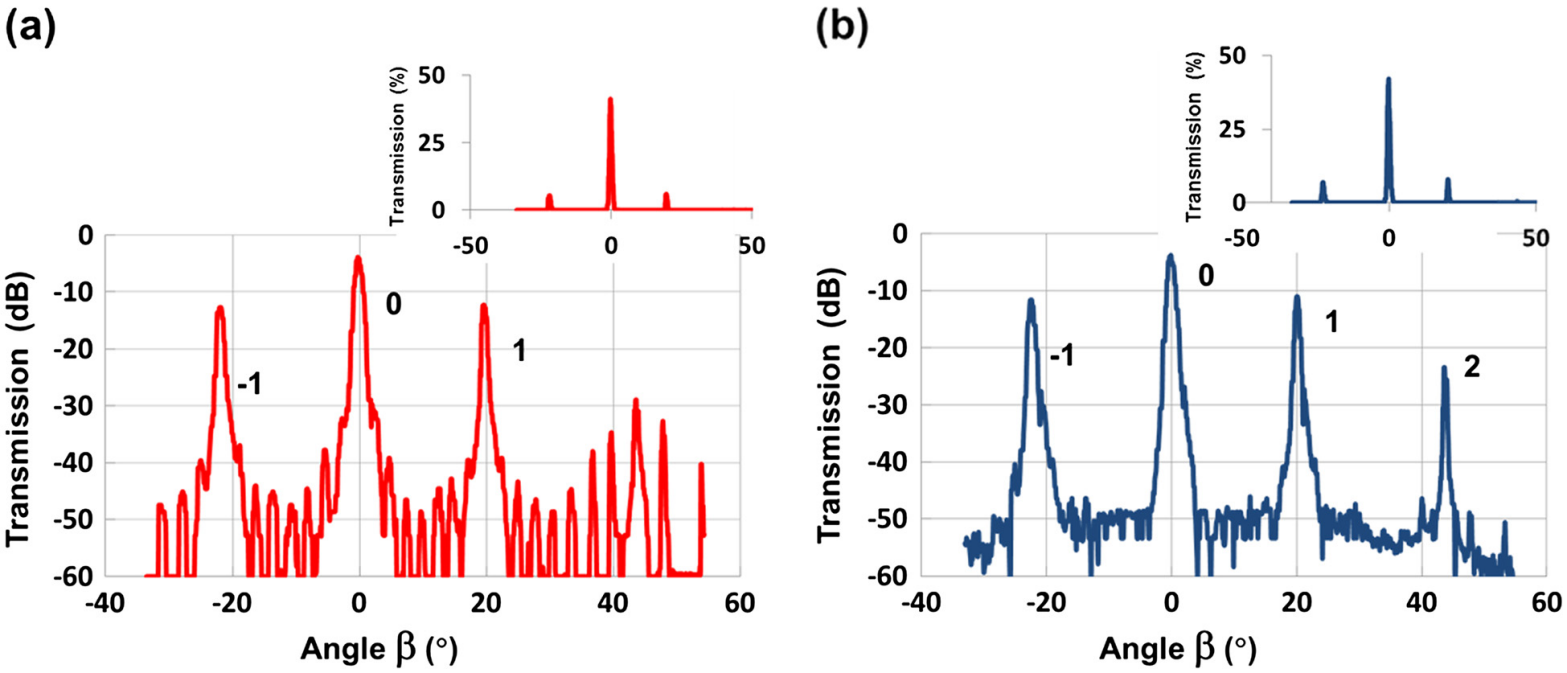
**Measured transmission data (logarithmic scale) of the diffraction orders at *λ *= 1,565 nm.** (**a**) Single-layer grating and (**b**) double-layer grating. The numbers next to the transmission peaks indicate the diffraction order. The inset in both plots shows the data on a linear scale.

Measurements at 1,565 nm of the diffraction efficiency transmitted *through* the porous grating and substrate (*α* = 4.5°) are shown in Figure 6a,b for the single- and double-layer gratings, respectively. The inset shows the same data on a linear scale. Key features of this data are the relatively few diffraction orders (compared to Figure 5), high transmission of >40%, and low scattering resulting in a background noise in the order of 10^−5^, which is 4 orders of magnitude lower than the 0th-order power. The high transmission is attributed to the optimized grating height which can be achieved using our processing techniques. The scattering losses are much lower at a wavelength of 1,565 nm compared to 632 nm, leading to significantly reduced noise where no diffracted orders are present. Both diffraction gratings produced similar diffraction order efficiencies; however, the background noise level at −50 dB for the single-layer grating showed fine scale structure not present in the double-layer grating. This may have been due to interferometric effects between the grating and the PS/silicon interface.

In our grating, the top layer was optimized to suppress the 0th-order transmission by choosing the top layer height as [8]:

(2) $$h_{1}=\frac{\lambda }{2\left(n_{1}-1\right)}$$

However, our patterned PS acts as both a phase and amplitude grating, and is formed on a high-index substrate which results in significant perturbation of the optical field through the structure. Such a complex, asymmetrical-layered structure does not obey simple models which predict 0th-order suppression with a 50% duty cycle grating [8]. The purpose of forming the *P*_2_ layer was to reduce reflections from the PS/silicon layer and improve the 0th-order suppression in transmission. This can be achieved using a layer of thickness:

(3) $$h_{2}=\frac{\lambda }{4n_{2}}$$

The *λ*/4 *P*_2_ layer introduces a *π* phase shift in the reflections from the interfaces either side of the *P*_2_ layer, suppressing the effect of these reflections in the transmitted beam (similar to an antireflection coating). By designing the layers as described by Equations 2 and 3, the 0th-order transmission through the grating can largely be suppressed.

**Figure 7:**
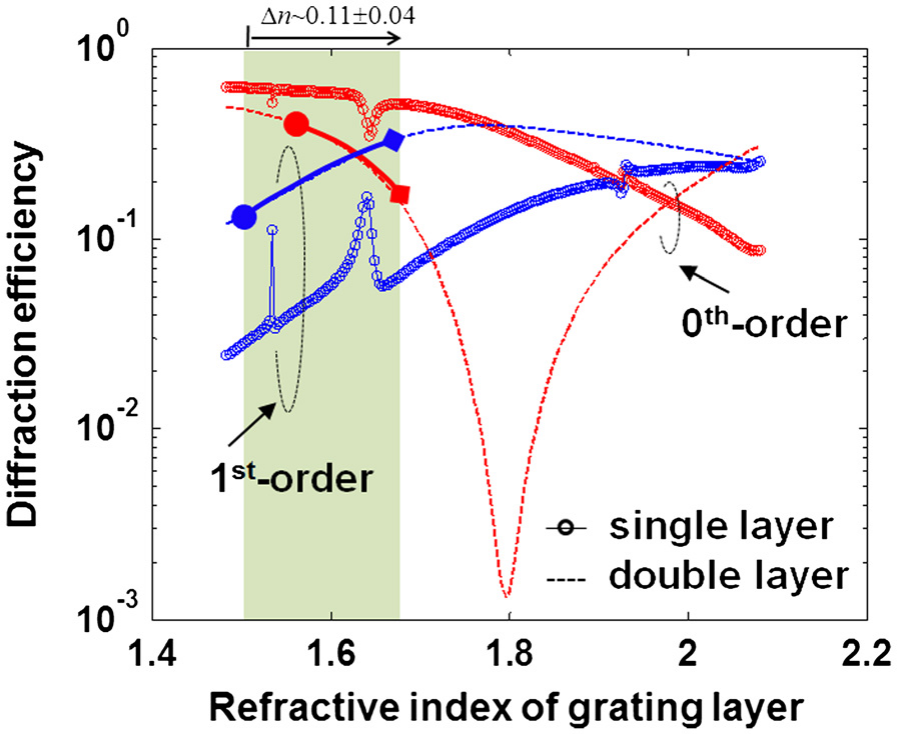
**Modeled 0th and 1st-order diffraction efficiencies as the index of the layer(s) is changed.** The single layer is drawn with a circle marker and the double layer gratings as a dashed line. Modeling was performed in transmission at a wavelength of *λ* = 1,565 nm and assumed no reflection from the backside of the silicon wafer.

A detailed understanding of this complex system requires a more thorough analysis. To understand the variation of diffraction efficiency with analyte induced index changes, a model based on the rigorous coupled mode theory [11-13] was evaluated by varying the index of the porous layers. The results are shown in Figure 7, as a function of the grating index layer and assuming no back reflection from the substrate backside. Comparing the model to the measured transmission in Figure 6a, the diffraction efficiencies of the single-layer grating matches well at the designed grating index layer of *n*_1_ = 1.78. For this grating, the diffraction efficiency variation with index shows several discontinuities which were attributed to the interferometric effects. Such changes are undesirable in a sensor where a monotonic response over the measurement range is required. For this grating, the 0th order is not suppressed for either the measurement or the model as predicted by Equation 1; this was due to the initial error in the design of the height (*h*_1_) of the single-layer grating. Nevertheless, good agreement with measurement validated the model and will enable subsequent optimization.

For the case of the double layer grating modeled in Figure 7, the *P*_2_ layer index was changed by the same amount as the grating *P*_1_ layer, which would occur when an analyte infiltrates both layers. The suppression of the 0th order is evident in the model at a refractive index of *n*_1_ = 1.78. However, the measured 0th-order diffraction efficiency for the double-layer grating shown in Figure 6b indicates that the 0th-order efficiency is higher than the 1st-order mode. The model in Figure 7 indicates this occurs at low-grating index values, suggesting that our estimated index for the fabricated layers is lower than expected. Nevertheless, the diffraction efficiency for the double-layer grating has a deeper 0th-order extinction and has a smoother transmission as index changes compared with the single-layer grating. Over an index range of 1.67 to 1.92, a minimum of 1 dB change in 0th-order diffraction efficiency occurs for a 1% change in normalize index change (*Δn*/*n*) - near the minimum of the transmission, up to 6 dB change for a 1% change in *Δn*/*n* occurs. These results indicate that a high extinction of the 0th order is important in achieving high sensitivity to changes in the refractive index. Separate modeling indicated that at a ridge/groove ratio of 39%, the minimum 0th-order diffraction efficiency for the double-layer grating, is reduced to more than 10^−4^, showing the importance of accurate patterning.

**Figure 8:**
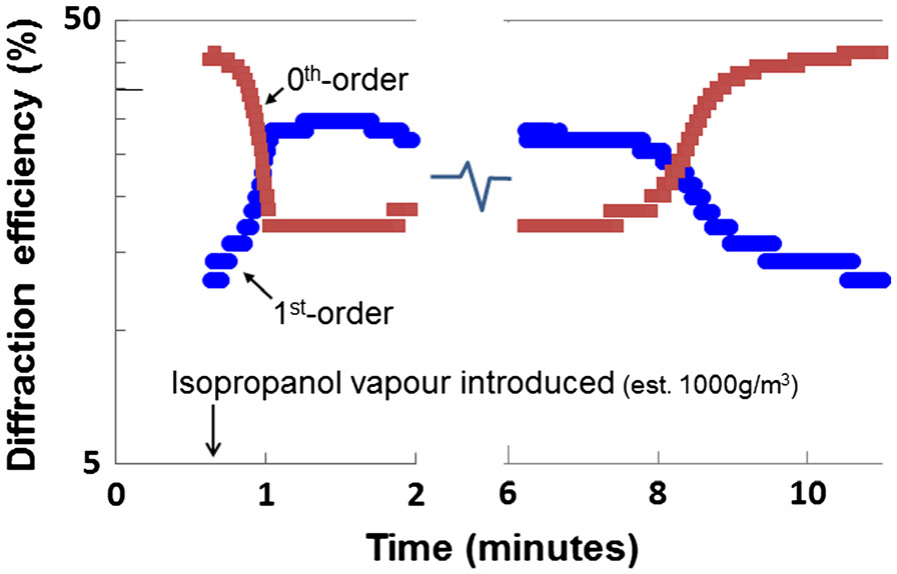
**Measured 0th-order and 1st-order diffraction efficiencies at *λ *= 1,565 nm after isopropanol vapor was introduced.**

To test the performance of our double-layer grating as a sensor, we simultaneously measured the 0th-order and 1st-order powers in transmission through the grating and substrate using the setup illustrated in Figure 2 at a wavelength of *λ* = 1,565 nm. The change in diffraction efficiency is shown in Figure 8, as isopropanol vapor was introduced to the surface at a concentration of 1,000 g/m^3^. Significant and rapid change in the diffraction efficiency was recorded as the vapor infiltrated the pores, resulting in the 1st-order diffraction efficiency becoming dominant, while the 0th order was suppressed. We were able to repeat these results many times, which demonstrated the reproducibility of the measurement. The results are consistent with the model for the double-layer grating shown by the shaded region in Figure 7, assuming the initial grating index was *n*_1_ = 1.53, and the index of the grating increased by *Δn* = 0.11 ± 0.04. This grating index is 14% lower than the design value of *n*_1_ = 1.78. The second anodization step is believed to be responsible for a 5% reduction in the index of the grating P_1_ layer, while the rest may have resulted from processing issues or film characterization errors requiring further investigation. The expected increase in grating index from the vapor, assuming pores saturated with isopropanol, is *Δn* = 0.3, suggesting only 35% saturation of the film by the vapor. The change in 0th-order and 1st-order efficiencies occurred within 30 s, largely due to the thin PS layers which the vapor needed to diffuse through to affect the refractive index within the pores. As the vapor dissipated (7 to 10 min), the diffraction efficiencies returned to their original values, indicating that the grating response was reversible and predictable. The steps observed in the data are a result of the sampled data quantization noise. An important feature of this highly sensitive nano-porous sensor is the complementary change in the 0th-order and 1st-order diffraction efficiencies. By measuring the difference between 0th-order and 1st-order diffraction efficiencies, the measurement sensitivity is improved, while common mode laser source noise is eliminated.

Improvements to the performance are expected from further modeling and design optimization. For the double-layer PS gratings with a high-index under-layer (*n*_2_), an antireflection layer could be formed under the grating [18] to increase the depth of observed null in the 0th order (Figure 7). High *Q* resonant waveguides could be fabricated using our methods to significantly enhance detection in PS sensors. For example, the fabrication of patterned features over layers of uniform index and thickness is a key to enable the formation of low loss layers required for resonant grating waveguides [19] and grating coupler waveguides [20]. While operating in the IR provides many advantages, one issue to contend with is the coherent interference that results when using a polished backside substrate. This interference is most predominant when the transmitted or reflected light is near an intensity minimum. Our modeling results indicate that the intensity dip in the transmitted 0th order shown in Figure 7 can vary from 10^−2^ to 10^−4^ as a result of coherent substrate reflections if the sample angle changes by as little as 0.1° relative to the incident light. Such uncontrolled variation could lead to significant errors in detection. These effects can be mitigated by either using broadband incoherent light sources or backside PS antireflection coatings which we have previously demonstrated [14].

In the sensors demonstrated here, the nanoscale pores within the films have been engineered in 2D, with the addition of highly uniform, nanometer-thick layers, and high-resolution microscale patterning of the films. These capabilities allow large, micrometer-sized cell and particle trapping between the gratings, while smaller nanometer-sized proteins and analytes could be captured and detected within the pores. The techniques described provide a path to combine both chemical [21] and physical [22] sensing in a single platform. Our process is capable of producing submicron features given suitable processing tools.

## Conclusions

This work has presented the model, design, and fabrication of multilayer diffraction gratings which have been optimized to operate in transmission at 1,565 nm. Operation at this wavelength reduces scattering losses and allows transmission through moderately doped substrates, enabling easier sample presentation. Moderately doped silicon ensures films containing nanometer-sized pores which allow large surface-area-to-volume ratios to be achieved. Passivation methods and polymer (ProLIFT) protection layers permit the use of standard photolithography and plasma etching techniques, typically used in the microelectronics industry. Our process allows a second (or more) anodization to be applied after the porous layer has been patterned. The second layer has uniform thickness and porosity, allowing antireflection coatings or waveguiding layers to be created, enhancing the sensing properties. Diffraction gratings with such under-layers demonstrate a large signal change as the refractive index of the medium around the grating changes, and the thin sensing layers enables rapid detection of analytes.

## Competing interests

The authors declare that they have no competing interests.

## Authors’ contributions

ML undertook all fabrication steps including porous silicon production and processes to produce the gratings. GMS and SB provided input to the models, provided the grating mask, and assisted with the design of the experiment. AK designed and undertook the optical measurements of the gratings, developed the grating models, and drafted the manuscript. GP provided guidance and input to the fabrication process and manuscript. All authors read and approved the final manuscript.

## Authors’ information

ML received the Bachelors in Electronic Engineering degree in 2004 from Chang'an University, Xi'an, China, and the Masters in Electronic Engineering in 2007 from the Northwestern Polytechnical University, Xi'an, China. She received her Ph.D. degree in 2012 from the University of Western Australia, Perth, Australia. Her main research interests are porous silicon and its applications for micromachining technologies. GMS obtained her M.Sc. in Applied Electronics from the National Institute of Technology, Tiruchirappalli in 2007. She is currently pursuing her doctorate under the guidance of Dr. Shanti Bhattacharya in the Department of Electrical Engineering, IIT Madras since 2012. Her current research interests include sub-wavelength structures and diffractive optics. GP (S’98-M’01) received the B.S. degree in Chemistry in 1995, and the bachelors and M.Sc. degrees in Electronic Engineering in 1995 and 1997, respectively, all from The University of Western Australia, Perth, and the Ph.D. degree in Electrical Engineering in 2001, from the University of California, Santa Barbara. She joined The University of Western Australia as an Australian Postdoctoral Fellow in 2001, and is now a professor at the same institution. Her main research interests are III-V nitride and porous silicon materials and devices. Specific interests within these areas currently include development of processing technology, transport studies, and development of novel chem- and bio-sensors. SB obtained her Ph.D. in Physics from the Indian Institute of Technology, Madras in 1997. Her Ph.D. work was in the area of optical array illuminators. She was awarded the Alexander von Humboldt award in 1998 and spent more than 2 years at the Technical University of Darmstadt, Germany. Her research work there included the development of an optical pick-up for CD/DVD systems and design of diffractive optical elements for beam shaping of high power laser beams. She subsequently joined the MEMS division of Analog Devices, Cambridge, USA, where she worked on the design of an optical MEMS switch. She is currently an associate professor and has been with the Department of Electrical Engineering, IIT Madras since 2005. Her current research interests are optical MEMS, diffractive optics, and fiber interferometry. AK received the bachelors and Ph.D. degrees in Electrical/Electronic Engineering in 1990 and 1995, respectively, from the University of Melbourne. He worked as a post-doctoral fellow at NTT (Musashinoshi, Japan) from 1996 and joined the UC Santa Barbara (USA) in 1998. He joined Calient Networks, Santa Barbara in 1999 as the Fiber Optics Technology Manager. In 2004, he joined the University of Western Australia as a research fellow, became an assistant professor in 2007, and a professor in 2010. He received the DSTO Eureka Prize for Outstanding Science in Support of Defence or National Security in 2008 for his contributions to the development of a MEMS microspectometer, and his current research interests include porous silicon for micromachined devices, optical MEMS biosensors, and microfluidics.

## References

[B1] Kemling JW Qavi AJ Bailey RC Suslick KS Nanostructured substrates for optical sensing J Phys Chem Lett 2011 2 22 2934 2944 10.1021/jz201147g 22174955 PMC3235654

[B2] Golub MA Hutter T Ruschin S Diffractive optical elements with porous silicon layers Appl Opt 2010 49 8 1341 1349 10.1364/AO.49.001341 20220890

[B3] Alexeev-Popov AV Gevelyuk SA Roizin YO Savin DP Kuchinsky SA Diffraction gratings on porous silicon Solid State Commun 1996 97 7 591 593 10.1016/0038-1098(95)00712-1

[B4] Rea I Iodice M Coppola G Rendina I Marino A De Stefano L A porous silicon-based Bragg grating waveguide sensor for chemical monitoring Sensors Actuators B-Chem 2009 139 1 39 43 10.1016/j.snb.2008.08.035

[B5] Ryckman JD Liscidini M Sipe JE Weiss SM Porous silicon structures for low-cost diffraction-based biosensing Appl Phys Lett 2010 96 17 171103-1 171103-3

[B6] Ruminski AM Manipulation of surface chemistry and nanostructure in porous silicon-based chemical sensors 2009 University of California: Ph.D. Thesis

[B7] Lerondel G Thonissen M Setzu S Romestain R Vial JC Holographic grating in porous silicon In Materials Research Society symposia proceedings 1997 452 631 636

[B8] Kress B Meyrueis P Digital Diffractive Optics: An Introduction to Planar Diffractive Optics and Related Technology 2000 Chichester, England: Wiley

[B9] Lai M Parish G Liu Y Dell JM Keating AJ Development of an alkaline-compatible porous-silicon photolithographic process J Microelectromech Syst 2011 20 2 418 423

[B10] Lai M Parish G Liu Y Keating AJ Surface morphology control of passivated porous silicon using reactive ion etching Microelectromech Syst J 2012 21 3 756 761

[B11] Harper KR Theory, design, and fabrication of diffractive grating coupler for slab waveguide 2003 Thesis: Brigham Young University

[B12] Moharam MG Grann EB Pommet DA Gaylord TK Formulation for stable and efficient implementation of the rigorous coupled-wave analysis of binary gratings J Opt Soc Am A 1995 12 5 1068 1076 10.1364/JOSAA.12.001068

[B13] Moharam MG Pommet DA Grann EB Gaylord TK Stable implementation of the rigorous coupled-wave analysis for surface-relief gratings: enhanced transmittance matrix approach J Opt Soc Am A 1995 12 5 1077 1086 10.1364/JOSAA.12.001077

[B14] James TD Keating AJ Parish G Faraone L Musca CA A technique for fabricating uniform double-sided porous silicon wafers Electrochem Solid-State Lett 2007 10 11 D130 D133 10.1149/1.2777007

[B15] Thonissen M Berger MG Billat S ArensFischer R Kruger M Luth H Theiss W Hillbrich S Grosse P Lerondel G Frotscher U Analysis of the depth homogeneity of p-PS by reflectance measurements Thin Solid Films 1997 297 1–2 92 96

[B16] Thonissen M Billat S Kruger M Luth H Berger MG Frotscher U Rossow U Depth inhomogeneity of porous silicon layers J Applied Physics 1996 80 5 2990 2993 10.1063/1.363156

[B17] Steiner P Lang W Micromachining applications of porous silicon Thin Solid Films 1995 255 1–2 52 58

[B18] Lee MSL Legagneux P Lalanne P Rodier JC Gallais P Germain C Rollin J Blazed binary diffractive gratings with antireflection coating for improved operation at 10.6 mu m Optic Eng 2004 43 11 2583 2588 10.1117/1.1802253

[B19] Rosenblatt D Sharon A Friesem AA Resonant grating waveguide structures IEEE J Quantum Electron 1997 33 11 2038 2059 10.1109/3.641320

[B20] Wei X Kang C Liscidini M Rong G Retterer ST Patrini M Sipe JE Weiss SM Grating couplers on porous silicon planar waveguides for sensing applications J Appl Phys 2008 104 12 123113-1 123113-5

[B21] Sweetman MJ Voelcker NH Chemically patterned porous silicon photonic crystals towards internally referenced organic vapour sensors RSC Adv 2012 2 11 4620 4622 10.1039/c2ra20232h

[B22] Birtwell SW Galitonov GS Morgan H Zheludev NI Superimposed nanostructured diffraction gratings as high capacity barcodes for biological and chemical applications Optics Commun 2008 281 7 1789 1795 10.1016/j.optcom.2007.04.066

